# High-Flow Nasal Cannula Oxygen Therapy in the Management of Respiratory Failure: A Review

**DOI:** 10.7759/cureus.50738

**Published:** 2023-12-18

**Authors:** Deyashini Mukherjee, Rahul Mukherjee

**Affiliations:** 1 General Internal Medicine, University Hospitals Coventry and Warwickshire, Coventry, GBR; 2 Respiratory Medicine and Physiology, Birmingham Heartlands Hospital, Birmingham, GBR; 3 Pulmonology, Institute of Clinical Sciences, University of Birmingham, Birmingham, GBR

**Keywords:** ltot, critical care, respiratory support, respiratory failure, niv, copd, hfnc

## Abstract

High-flow nasal cannula (HFNC) oxygen therapy is gaining traction globally as a treatment for respiratory failure. There are several physiological benefits, and there is a growing body of evidence showing improved quality of life and patient comfort with HFNC, both in acute and home settings. Due to the increased burden of long-term respiratory conditions such as chronic obstructive pulmonary disease (COPD) on healthcare systems worldwide, the role of ward-based and post-discharge interventions in the prevention of hospital readmissions is an area of increasing interest. In this narrative review, we outline the physiological effects of HFNC and assess its applications in both the hospital and home settings for acute and chronic respiratory failure. We also consider the evidence of non-invasive ventilation (NIV) versus HFNC in the hospital setting and the application of HFNC at home in stable hypercapnic respiratory failure to improve the quality of life and prevent readmissions. We also look at applications of HFNC in specific circumstances, such as the perioperative period, emergency department, and acute (mainly critical care) setting including in immunocompromised patients and palliative care.

## Introduction and background

**Figure 1:**
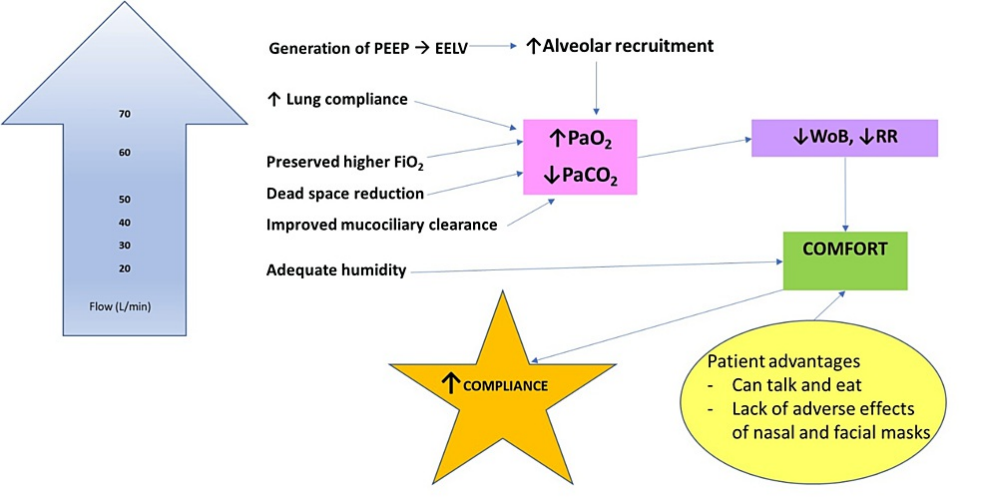
Schematic representation of the physiological effects of HFNC in respiratory failure L/min: Liters per minute; FiO_2_: Fraction of inspired oxygen; PEEP: Positive end-expiratory pressure; EELV: End-expiratory lung volume; PaO_2_: Partial pressure of oxygen in arterial blood; PaCO_2_: Partial pressure of carbon dioxide in arterial blood; WoB: Work of breathing; RR: Respiratory rate. Image credits: Authors of this study.

High-flow nasal cannula (HFNC) oxygen therapy is being used globally in a wide variety of settings as a treatment for both acute and chronic respiratory failure. In the acute setting, it can be used as a type of respiratory support therapy in addition to oxygen (O_2_) delivery via simple facemask and non-invasive ventilation (NIV). While there is growing evidence of the utility of HFNC in acute hypoxemic respiratory failure, the evidence regarding its efficacy in acute hypercapnic respiratory failure is sparse. The European Society of Intensive Care Medicine’s 2023 guidelines on the management of acute respiratory distress syndrome recommend the use of HFNC over conventional oxygen therapy in non-mechanically ventilated patients to avoid the risk of intubation except cardiogenic pulmonary edema and acute exacerbation of chronic obstructive pulmonary disease (COPD) [1]. HFNC can deliver high flows of O_2_ with rates of 20-70 L/minute with a fraction of inspired O_2_ (FiO_2_) ranging from 0.2 to 1.0 [2]. There are differential physiological benefits at different rates of HFNC delivery. In the intermediate range of around 20-45 L/min, there is improvement in work of breathing, improved mucociliary clearance, and dead space washout, while at the higher range of around 60-70 L/min, it increases airway pressure, end-expiratory lung volume, and oxygenation [3,4]. These mechanisms improve pulmonary mechanics and lead to improved clinical parameters: reduced respiratory rate and work of breathing. This in turn improves patient comfort. Increased patient comfort enables patients to comply with therapy for a longer duration (Figure 1).

Setup and delivery

While HFNC is still predominantly used in the critical care setting, it is easy to set up on medium- to high-dependency wards and even at home. There are four essential components of the HFNC circuit: flow generator (air/oxygen blender, turbine, or Venturi mask), active heated humidifier unit, heated tubing and chamber kit, and lastly the nasal cannula interface. The nasal cannulae come in a range of sizes to account for anatomical differences in facial structure. In the hospital setting, the device would have to be set up with an oxygen source and a high-pressure air source. Devices have been adapted to use a turbine instead of a domiciliary setting. The flow rate, FiO_2_, and temperature can all be set and adjusted to optimize patient comfort. The ranges and upper limits of all these factors are device-dependent. Flow rates can typically be up-titrated to 60-70L/min. This should match the patient’s inspiratory flow rate [4,5]. This avoids inhalation of excess ambient air, hence maintaining a stable FiO_2_ to the alveoli without dilution [4,5]. However, dilution may be unavoidable for patients in acute respiratory distress with higher flow rates up to and over 100L/min, where maximal flow rate achievable on the device should be selected [4]. Basile et al. showed that when treating acute hypoxemic failure with HFNC, flow rates between 75 and 120 L/min were associated with reduced respiratory rate, increased ventilation homogeneity, and increased positive pressure effect but with poorer patient comfort [6]. FiO_2_ can be adjusted between 60% and 100% through the oxygen inlet port. The in-built oxygen sensors can analyze the FiO_2_ of the inspired gas, therefore minimizing discrepancies between the setting and the FiO_2_ delivered to the patient [3]. Most manufacturers recommend setting the temperature to around 37°C to achieve a relative humidity of 100% [7], thereby maximizing patient comfort. Devices will typically be able to set temperatures between 31°C and 39°C, with a setting below the patient’s core body temperature depending on patient preference [4]. Currently, Airvo2 humidification equipment and Optiflow interface system (Fisher and Paykel, Maidenhead, UK) are two of the most frequently used HFNC devices in the United Kingdom. Nishimura provided further head-to-head device descriptions in the 2019 review [8].

Physiological effects of HFNC

There are many physiological benefits of HFNC. First, HFNC improves dead space washout. The anatomical dead space consists of segments of the respiratory tract where air is conducted to alveoli, but there is no exchange of O_2_ and CO_2_ across the alveolar membrane. In normal ventilation, around 30% of the tidal volume is wasted due to dead space. In COPD, an increase in dead space leads to impaired ventilation. Delivery of high-flow gas has been shown to improve ventilation [9]. HFNC is delivered via nasal cannulae, creating an oxygen reservoir by allowing continuous flushing of exhaled gases in the upper airways [10]. As the flow is increased, the dead space washout improves, thereby improving gas exchange and decreasing the PaCO2m [4,11]. Thus, the dead space washout effect is particularly beneficial, especially as compared to NIV, which increases anatomical dead space with the use of the tight-fitting face mask [11].

Second, HFNC improves the work of breathing and pulmonary mechanics. Work of breathing is defined as the energy expended to produce a constant tidal volume (TV) over a unit of time. This is dependent on lung compliance and airway resistance. Clinically, increased work of breathing translates to signs such as nasal flaring and the use of abdominal muscles. In respiratory distress, HFNC reduces the respiratory rate [12]. HFNC also improves thoracoabdominal synchrony as compared to conventional oxygen therapy in patients with mild to moderate respiratory distress [13]. Therefore, in theory, HFNC reduces the work of breathing. Clinically, HFNC has been shown to decrease the work of breathing head-to-head against conventional oxygen therapy [14]. The electrical activity of the diaphragm significantly decreased during the post-extubation phase in acute exacerbation of COPD with HFNC versus conventional oxygen therapy [15]. Another study assessed diaphragm activation using ultrasound in patients recovering from acute hypercapnic respiratory failure treated with NIV. The study showed that after NIV interruption, diaphragm activation increased with conventional oxygen therapy but not with HFNC [16]. This positive effect on the work of breathing may render HFNC sufficient in hypercapnic patients to avoid escalation to NIV [11]. Although HFNC reduces minute ventilation by reducing the respiratory rate, it improves TV compared to long-term oxygen therapy (LTOT) in healthy volunteers with stable COPD and in critical care patients [17]. Therefore, due to dead space washout, alveolar recruitment, and improvement in TV, HFNC improves the overall efficiency of ventilation [11].

Third, HFNC improves mucociliary clearance. The respiratory epithelium is very sensitive to changes in airway temperature and pressure. It functions best at core body temperature and 100% relative humidity [4]. A fall in temperature can affect the consistency of respiratory secretions and reduce cilia beating, thereby reducing the speed of mucociliary clearance [4,7]. Administration of conventional oxygen therapy, a cool dry gas, alters the physical and biochemical properties of respiratory mucus and can precipitate bronchoconstriction. This is worse in patients with established airway diseases such as COPD. Reduced mucociliary clearance increases the chances of mucus plugging, worsening infections, and eventual lung function decline [4,18]. Effective tracheobronchial secretion clearance occurs when the inhaled air is fully saturated and conditioned to 37°C in patients with obstructive airway disease [19]. Therefore, the heated humidification built into HFNC circuits improves patient comfort and concordance by improving mucociliary clearance and maintaining comfortable levels of moisture in the airways.

Limitations and contraindications

While there are many advantages of HFNC, there are also some limitations. The first few minutes after initiation of HFNC might still be uncomfortable, but this is circumvented by gradual up-titration of the flow rate to the desired level to allow patients to become accustomed to HFNC and endure the therapy for a longer period [4]. There is also a risk of skin breakdown, but this is less significant than seen with NIV [4,20]. The mask fit may still be an issue, which causes leaks, resulting in a less effective positive airway pressure effect [21]. HFNC is more expensive as compared to conventional oxygen therapy [21]. In addition, it is an intervention with its own intricacies that require additional training for the multidisciplinary team in the ward and critical care settings to initiate HFNC therapy. Furthermore, HFNC may delay intubation and may also delay time-critical discussions regarding ceilings of care or treatment, resuscitation, and making end-of-life decisions [21,22].

Contraindications of HFNC include altered level of consciousness including severe agitation, airway obstruction, risk of aspiration, facial injury (particularly nasopharyngeal trauma or recent surgery), respiratory arrest, hemodynamic instability, excess sputum, and claustrophobia [23]. Table 1 shows a comparison of HFNC versus other modes of respiratory support.

**Table 1:** Comparison of different methods of respiratory support PEEP: Positive end-expiratory pressure; HFNC: High-flow nasal cannulae; NIV: Non-invasive ventilation.

Type of respiratory support	Benefits	Limitations	Complications
Conventional oxygen therapy	Easy to use, no additional training required and accessible everywhere, no delay in initiation, and patients can eat and drink while therapy is ongoing	No humidification and limited patient comfort due to mouth dryness	Possible over-oxygenation
High-flow nasal cannulae	Heating and humidification improve patient comfort and compliance, PEEP effect, washout of dead space, mucociliary clearance, decreased work of breathing, and patients can eat and drink while therapy is ongoing	Not always available in all settings and requires additional training	Possible over-oxygenation and delay in intubation
Non-invasive ventilation	Greater PEEP effect than HFNC, alveolar recruitment, reduction of left ventricular afterload, and reduced respiratory muscle workload	Reduced patient comfort due to tight-fit mask and no humidification, increased dead space, requires additional training, and patients cannot eat and drink while therapy is ongoing	Pneumothorax, gastric distension, aspiration pneumonia, pressure ulcers, increased skin breakdown of the nasal bridge and cheeks, hemodynamic instability, and delay in intubation
Invasive mechanical ventilation	All benefits of HFNC and NIV, control of parameters, and in-built alarms	Specialist skills therefore only available in the presence of an anesthetist/intensivist	Lung or airway injury during the procedure, esophageal intubation, dislodgement of the endotracheal tube, ventilator-associated pneumonia, sepsis, pneumothorax, aspiration, and hemodynamic instability

## Review

Clinical applications

There are many established clinical applications of HFNC and possible applications in the future based on physiological effects (Table 2).

**Table 2:** Summary of established and potential applications of HFNC in suitable settings and physiological effects ED: Emergency department; ICU: Intensive care unit; HDU: High-dependency unit; COPD: Chronic obstructive pulmonary disease; FiO_2_: Fraction of inspired oxygen; PaO_2_: Partial pressure of oxygen in arterial blood; PaCO_2_: Partial pressure of carbon dioxide in arterial blood; WoB: Work of breathing; NIV: Non-invasive ventilation.

Clinical application	Setting of application	Physiological effects
Acute respiratory failure	ED, ICU, hospital ward	Maintains higher FiO_2_, improves lung compliance, improves alveolar recruitment, increases PaO_2, _decreases PaCO_2_, reduces WoB, improves patient comfort
Acute exacerbation of COPD	ED, ICU, HDU, hospital ward	Increases dead space washout, leading to improved gas exchange and reduction in PaCO_2_
Stable hypercapnic COPD	Home	Reduces exacerbations and PaCO_2_, leading to improved quality of life
Support during exercise in COPD	Home, pulmonary rehabilitation	Improves oxygenation, leading to less dyspnea, leading to increased exercise tolerance
Cardiogenic pulmonary edema	ED, ICU, HDU, hospital ward	Improves oxygenation and decreases afterload
Prevention of reintubation	Theaters, ICU, HDU, hospital ward	Improves gas exchange similar to NIV
Preoxygenation during airway procedures	Theaters	Increased apnea time
Breaks from positive airway pressure	ICU, hospital ward	Patients can eat and talk
Immunocompromised patients	ED, ICU, hospital ward	Improves gas exchange similar to NIV
Palliative patients	ED, hospital ward, ICU/HDU if clinically appropriate, home, hospice	Decreases breathlessness
Bronchiectasis and cystic fibrosis	ICU, hospital ward, home	Improves mucociliary clearance, leading to improved ventilation
Prehospital care and patient transfer	Prehospital, ambulance	Maintains higher FiO_2_, improves lung compliance, reduces WoB, improves patient comfort

Acute Hypoxemic Respiratory Failure

It is commonly used in the treatment of hypoxemic respiratory failure, which is caused by a ventilation/perfusion mismatch, hypoventilation, limitation of diffusion, right-to-left shunt, and reduced inspired oxygen tension [4]. Conventional oxygen therapy can be administered via nasal cannulae, with flow rates up to 5-6 L/min, via simple facemask up to about 10 L/min, and via non-rebreathe mask with rates of 15-20 L/min. While oxygen is the treatment, conventional oxygen therapy is often not sufficient to keep up with the demands of patients in hypoxemic respiratory failure, who have flow rates up to about 60 L/min [4]. Prolonged treatment with conventional oxygen therapy without humidification can also lead to adverse effects such as dry mouth, increased upper airway secretions, and epistaxis [4,24].

The use of HFNC in acute hypoxemic respiratory failure should be closely monitored to avoid delay in intubation when it is required [3]. The ROX index (ratio of oxygen saturation as measured by pulse oximetry/FiO_2_ to respiratory rate) is used to serially assess the need to either escalate oxygen therapy or intubate promptly in patients with hypoxemic respiratory failure [3]. The advantage of the ROX index is that it looks at the best describers of the patient’s respiratory status in a single figure [3]. However, it is important to remember that the ROX was validated in acute hypoxemic respiratory failure related to pneumonia [3]. Therefore, adjustments may need to be made depending on the underlying cause of respiratory failure. Ricard et al. outlined the guideline ROX index cut-offs at 2, 6, and 12 hours post-initiation of HFNC therapy to aid decision-making for escalation of treatment [3]. The authors suggested monitoring the current treatment for a ROX index above 4.88 at all three time points. On the other hand, if the ROX index is less than 2.85, 3.47, and 3.85 at 2, 6, and 12 hours post-initiation of HFNC therapy, respectively, endotracheal intubation should be considered. For an interim ROX index at each time point, HFNC support should be increased followed by reassessment in 30 minutes. Furthermore, they showed that patients who went on to require endotracheal intubation had smaller increases in their ROX index between 2 and 12 hours as well as between 6 and 12 hours [3]. This further reinforces the need for ongoing adaptive assessment of the ROX index to identify patients who are likely to progress to endotracheal intubation earlier [3].

Although not widely used clinically, HFNC may be effective in the management of cardiogenic pulmonary edema. Guidelines advocate the use of positive airway pressure to improve oxygenation and decrease cardiac afterload. Studies have shown that HFNC can achieve the same physiological effects with less discomfort for the patient [25,26]. Makdee et al. showed that HFNC may decrease the severity of dyspnea during the first hour of treatment. However, they did not demonstrate any statistically significant difference in admission rate, emergency department and hospital lengths of stay, NIV, or intubation [26]. More randomized controlled trials (RCTs) looking specifically at patients with cardiogenic pulmonary edema as the cause of hypoxemic respiratory failure are needed to adopt HFNC as part of its management guidelines.

Acute Hypercapnic Respiratory Failure

As previously mentioned, HFNC is effective in managing acute exacerbations of COPD by improving washout of dead space, therefore ameliorating gas exchange and leading to a reduction in PaCO_2_. Pisani et al.’s 2019 systematic review [14] found that HFNC can keep PaCO_2_ unmodified, while oxygenation slightly deteriorates as opposed to NIV. Furthermore, the work of breathing is reduced with HFNC to a similar extent to NIV. HFNC is also reported to be more comfortable than conventional oxygen therapy and NIV. The review concluded that there was little and limited evidence for improved clinical outcomes [14].

HFNC is also a useful support device to allow patients to eat, drink, and talk while taking breaks from positive airway pressure, which is commonly used to treat many causes of respiratory failure in a variety of hospital settings [4].

HFNC Versus NIV in Acute Respiratory Failure

HFNC and NIV are both established forms of treatment for acute respiratory failure. There are an increasing number of head-to-head studies looking at the effectiveness of HFNC compared to NIV. Lee et al. conducted a systematic review of 12 studies comparing HFNC versus NIV (and conventional oxygen therapy) in terms of oxygenation, breathing mechanics, escalation of oxygen therapy, mortality, and patient comfort [27]. HFNC was inferior to NIV for oxygenation [27]. HFNC did not significantly reduce respiratory rate in two studies [27-29]. In Frat et al.’s 2015 study, there was a significantly reduced respiratory rate after one hour of HFNC therapy but not after six hours of HFNC therapy [27,30]. They also showed that HFNC significantly reduced intubation rate in those with baseline PaO_2_:FiO_2_ ≤ 200 mmHg (P/F ratio) as compared to NIV (35% intubation rate in HFNC with 95% CI: 25%-46% versus 58% in NIV with 95% CI: 47%-68%, p = 0.009) but not above this P/F ratio threshold [27,30]. Frat et al. showed that both 90-day mortality and ICU mortality were significantly reduced in those who received HFNC as compared to those who received NIV [27,30]. Several studies showed that HFNC was significantly more comfortable than conventional oxygen therapy [27]. In a few studies looking at comfort head-to-head HFNC versus NIV, dyspnea significantly improved with HFNC [27,30], although some studies did not find the difference to be statistically significant [27].

Sun et al. performed an observational cohort study of 82 COPD patients with acute hypercapnic respiratory failure in 2019, comparing HFNC and NIV as the initial treatment in intensive care [31]. This showed no significant difference in treatment failure, intubation rates, length of ICU stay, or 28-day mortality between the two groups [31]. However, there were significantly fewer nursing airway care interventions, increased device application time, and significantly fewer episodes of skin breakdown in the HFNC group as compared to the NIV group [31].

Feng et al. more recently conducted a systematic review including eight studies, looking specifically at HFNC versus NIV in AECOPD patients post-extubation [32]. Their primary outcome was the reintubation rate. They found that in those with hypercapnia, there was no statistically significant benefit of HFNC over NIV [32]. However, in those without hypercapnic respiratory failure, there was a statistically higher reintubation rate in the HFNC group as compared to NIV [32]. Feng et al. looked at mortality, ICU length of stay, complication rates, and a handful of other assorted outcomes as secondary outcomes [32]. There was no significant difference in mortality or length of stay in the ICU in the HFNC group as compared to NIV, regardless of hypercapnia [32]. However, HFNC significantly reduced complication rates (nasal facial skin breakdown, aspiration, and flatulence during treatment) in AECOPD patients post-extubation in all patients [32]. There was no significant difference in heart rate, PaCO_2_ post-extubation, pH, or P/F ratio in any of the patients regardless of hypercapnia [32]. However, there was significantly reduced RR in patients with hypercapnia but not in the non-hypercapnic group [32].

Bruni et al. concluded that in the case of respiratory acidosis, NIV remains the gold-standard treatment. However, HFNC may be considered as an alternative to NIV if the latter fails for intolerance. HFNC should also be considered and preferred to conventional oxygen therapy at NIV breaks and weaning. Finally, HFNC should also be preferred to conventional oxygen therapy as first-line oxygen treatment in AECOPD patients without respiratory acidosis [33].

Overall, the literature suggests that HFNC is non-inferior to NIV in the management of acute hypercapnic failure with regard to intubation rates, escalation of oxygen therapy, length of stay in ICU, and mortality. HFNC appears to be superior in terms of patient comfort and reduces complications of therapy. However, caution should be exercised in hypercapnic AECOPD as there is some evidence of worsening hypercapnia with higher FiO_2_ for unchanged flow rates [34]. Further RCTs with large sample sizes are required to explore the direct comparison of HFNC versus NIV, especially with the advancement of both the nasal cannulae and high-flow oxygen delivery systems.

HFNC in Prehospital Care and Patient Transfer

Due to the myriad of benefits of HFNC over conventional oxygen therapy and advantages over both non-invasive and invasive ventilation, HFNC is a suitable method of oxygenation in the context of prehospital care and during patient transfer. Several studies have looked at the use of HFNC in the transfer of pediatric and neonatal patients [35,36]. Al-Mukhaini and Al-Rahbi concluded that HFNC was safe in the non-critical care pediatric setting, provided that there was enough expert supervision and monitoring [35]. Reimer et al. found that HFNC was not only effective in the pediatric population but also in adults, with no evidence of physiological decompensation post-transfer [37]. Therefore, it is a feasible additional option for oxygenation. Further work needs to be done to see whether HFNC is superior or non-inferior to NIV and other non-invasive methods of oxygenation in terms of safety and patient-reported comfort and to ascertain sensible limits of when HFNC can be used in the context of patient transport. A case report outlined a successful helicopter transfer of a patient with end-stage lung disease to a transplant center in Lisbon [38]. This is promising and should be explored further as an additional method of oxygen in comparison to NIV. Inkrott and White also postulated that HFNC is likely to become a mainstay of air transport, given its relative ease of setup and apparent similar outcomes as compared to NIV in terms of reducing the need for intubation, therefore highlighting the need to incorporate HFNC as part of the air transport programs with adequate pre-transfer planning of equipment and logistics [39]. Overall, HFNC is safe and effective in the out-of-hospital setting and pediatric interfacility patient transfers, but more work needs to be done to determine the most suitable patient groups who will benefit from HFNC in the prehospital context [40].

HFNC in the Emergency Department

Respiratory failure is a very common presentation to the emergency department, where it may be postulated that HFNC could have an important role in its treatment. However, Tinelli et al.’s 2019 systematic review and meta-analysis looked at four RCTs comparing HFNO to conventional oxygen therapy exclusively in ED, where HFNC did not improve intubation requirement, treatment failure, hospitalization, or mortality [41]. In one RCT looking at HFNC versus NIV, there was no difference in intubation requirement, treatment failure, tolerance, or dyspnea [41,42]. This could be due to the underlying reason for respiratory failure and rate of deterioration as well as the type of and familiarity with the equipment. In Australia, Bell et al. conducted an RCT, which found that in selected patients with undifferentiated cause of shortness of breath, there was an association with improved respiratory rate two hours post-initiation of HFNC treatment and also lower escalation in ventilation strategy as compared to conventional oxygen therapy [43]. Rittayamai et al. concurred that HFNC improved dyspnea and comfort in subjects presenting with acute dyspnea and hypoxemia in the emergency department [44]. However, the HOT-ER study concluded that HFNC did not reduce the need for mechanical ventilation in the emergency department for subjects with acute respiratory distress compared to conventional oxygen therapy [45]. They found that HFNC was safe and may reduce the need for escalation of oxygen therapy in the first 24 hours of admission [45]. The data looking at the use of HFNC in the emergency department is very limited, and much more work needs to be done to determine its effectiveness.

Use of HFNC in COVID-19-Related Respiratory Failure

HFNC was widely used in acute hypoxemic respiratory failure due to coronavirus disease 2019, although the data is limited. The RECOVERY-RS trial found no significant difference between an initial strategy of HFNC compared to conventional oxygen therapy [46]. However, a retrospective study in patients not mechanically ventilated within six hours of admission in the five hospitals of the Johns Hopkins Health System, USA, found that HFNC was associated with a significantly reduced hazard of death [47]. Schmid et al. conducted a systematic review and meta-analysis of five RCTs, which concluded that neither HFNC nor NIV reduced mortality in COVID-19 patients in critical care units. Both HFNC and NIV carried a substantial risk of harm in such patients. Although the evidence was not compelling, the use of NIV tended to reduce the need for endotracheal intubation as compared with HFNC [48]. Liu and Cheng’s review postulated that HFNC could decrease the risk of intubation and provide satisfactory patient comfort in COVID-19-related respiratory failure. HFNC could also enable resource allocation, allowing ventilators to be reserved for the most severely ill patients. They, however, observed that close monitoring using the ROX index or other respiratory parameters was essential to avoid a delay in endotracheal intubation if clinically indicated [49].

HFNC in the Perioperative Setting

HFNC has several roles in different aspects of the perioperative period. HFNC is a promising tool in preoxygenation in patients, especially when used during awake fiber-optic intubation or in patients with difficult anticipated intubation [3,50]. Moreover, patients with hypoxemic respiratory failure due to COPD or other underlying illnesses (idiopathic pulmonary fibrosis, severe obesity, neuromuscular disorders, or pregnancy) are more prone to severe oxygen desaturation. This is because the oxygen stores are depleted by increased oxygen consumption [50]. NIV can be used for preoxygenation as it reduces alveolar collapse and the likelihood of atelectasis, which leads to hypoventilation, thereby increasing the ventilation/perfusion mismatch [50]. However, NIV cannot be used during laryngoscopy and, therefore, cannot prevent desaturation during tracheal intubation [50]. HFNC has an advantage over NIV in laryngoscopy as it is delivered purely via nasal cannulae [50]. HFNC increases apnea time with adequate oxygenation without hypercapnia [50-52]. Evidence shows that in patients with moderate to severe hypoxemia (PaO_2_/FiO_2_ ≤ 200mmHg), desaturation to <80% occurred significantly less frequently after preoxygenation with NIV as compared with HFNC (24% versus 35%, p = 0.046) [3,53]. Another study showed that the combination of both NIV and HFNC significantly reduced significant desaturation during intubation as compared to NIV alone [54]. Based on this, Ricard et al. suggested that preoxygenation with HFNC may be considered in patients with a PaO_2_:FiO_2_ ratio (P/F ratio) between 200 and 300 mmHg, while NIV may be superior at lower P/F ratios [3].

The role of HFNC post-surgery is more complicated and requires further evaluation. Generally, surgical patients are classed as having a low risk of needing reintubation due to generally shorter periods of mechanical ventilation [3]. Other factors, such as anesthetic factors and surgical complications, may render surgical patients at higher risk [3]. There is evidence to suggest that HFNC post-extubation in low-risk patients, including complex surgical patients with >12 hours of mechanical ventilation, reduces the reintubation rate as compared to conventional oxygen therapy [3,50,55]. HFNC was also shown to be non-inferior to NIV [32]. However, the data was very heterogeneous [3,50]. Other studies have not found such differences in uncomplicated post-abdominal surgery patients [3,56]. Zhu et al. conducted a meta-analysis, which confirmed that HFNC was superior to conventional oxygen therapy in managing post-extubation respiratory failure and avoiding reintubation following planned extubation [57].

HFNC is also useful in specific respiratory procedures such as elective and emergency bronchoscopy, especially in patients with pre-existing chronic respiratory disease. HFNC has been shown to improve oxygen throughout the procedure as compared with conventional oxygen therapy [4,58].

Frat et al. found that patients with a P/F ratio less than 200 mmHg had a lower intubation rate when treated with HFNC as compared to patients treated with NIV or conventional oxygen therapy [30]. Consequently, there was a clinically relevant difference in terms of reduction in hospital and 90-day mortality rate for patients who had received HFNC as first-line treatment [30].

HFNC in Immunocompromised Patients

There is unclear evidence regarding whether HFNC is beneficial in the management of acute hypoxemic respiratory failure in immunocompromised patients. This is due to the heterogeneity of the patient population and the cause of immunosuppression. This may be iatrogenic, for example, due to steroids or chemotherapy medications. It could also be due to the underlying disease, for example, a hematological malignancy or a primary immunodeficiency [4]. Ricard et al. reviewed several studies and concluded that HFNC may reduce the intubation rate as compared to HFNC in immunocompromised patients [3]. Azoulay et al. showed that there were no significant differences in 28- or 90-day mortality, ICU stay, intubation rates, or breathlessness between those treated with HFNC versus those treated with conventional oxygen therapy via facemask [4,59]. The multivariate analysis confirmed that there was a trend toward lower intubation rates in the HFNC group [3,59]. The results could partly be due to overall lower survival rates in immunocompromised patients as compared to immunocompetent patients [4,60]. Causes of lower survival rates could be due to the propensity of immunocompromised patients to develop multisystem complications of their underlying disease or pharmacological therapies. Utilization of HFNC in immunocompromised patients should occur with the input of a multidisciplinary team to simultaneously optimize the management of comorbidities, correct acute respiratory failure, and enable prompt recognition and treatment of any complications [4].

HFNC at Home

Advanced COPD leads to significantly compromised exercise tolerance and progressive inability to carry out activities of daily living. They are prone to exacerbations, associated with hospitalization and increased mortality. After smoking cessation and pulmonary rehabilitation programs, LTOT is beneficial for the longer-term survival of COPD patients with severe resting hypoxemia. Established criteria exist for starting LTOT:PaO_2 _< 7.3 kPa on room air or PaO_2_ < 8.0 kPa with one of secondary polycythemia (hematocrit > 55%), right heart failure, or cor pulmonale [61].

HFNC is an emerging treatment in the management of chronic hypercapnic COPD. Storgaard et al. compared LTOT + HFNC with LTOT alone in 200 patients [62]. They showed that HFNC significantly reduced AECOPD rates as compared to LTOT alone (3.12 versus 4.95 exacerbations/patient/year, p < 0.001), but there was no significant difference in the annual hospital admission rate. The HFNC group had significantly fewer exacerbations in the study year as compared to the previous year (p < 0.001). Dyspnea as calculated by the mMRC score was significantly better at three months and onward in the HFNC group as compared to the LTOT group. Quality of life (QoL) as calculated using the St. George’s Respiratory Questionnaire (SGRQ) was better at 6 and 12 months in the HFNC group [29,62]. PaCO_2_ gradually decreased and was significantly lower in the HFNC group at 12 months. Six-minute walk test (6MWT) distances were significantly better in the HFNC group at 12 months. Although not statistically significant, there was a trend toward increased FEV1 in the HFNC group as compared to LTOT at 6 and 12 months. There was no difference in the FVC%, FEV1/FVC, PaO_2_, SaO_2_, or pH between the two groups at 6 or 12 months. Interestingly, there was an increase in BMI in the HFNC group as compared to the LTOT group [62].

Nagata et al. performed a multicenter crossover trial in 2018, where patients with stable hypercapnic respiratory failure with Global Initiative for Chronic Obstructive Lung Disease (GOLD) classification 2-4 (2013 guidelines) [63] already receiving LTOT for at least one month received LTOT for six weeks, followed by LTOT + HFNC for six weeks and vice versa [64]. The study was recruited from nine hospitals in Japan. The total SGRQ-C (compact SGRQ) score, as well as all its individual components (symptoms, activity, and impact) reflecting QoL, significantly improved in those receiving HFNC. Furthermore, PaCO_2_, pH, and ptCO_2_ significantly improved in the HFNC group [64]. There was no significant difference in 6MWT (distance, SpO^2^ decline %, and Borg scale for dyspnea), pulmonary function tests (vital capacity, FVC, FEV1, FEV1/FVC, DLCO, RV, FRC, and TLC), or physical activity (calorie consumption, step count, and activity time) [64]. They also studied the impact of HFNC on health economics by studying quality-adjusted life years (QALYs) using the five-level version of the EuroQol five-dimensional questionnaire (EQ-5D-5L) score [64,65]. They found that there was no statistically significant difference in QALYs between HFNC + LTOT and LTOT alone [64], although there was a statistical difference when using the visual analog scale version of the same scoring system [64]. They found that the net difference equated to a benefit of 15,780 Japanese Yen (US$158.70) per patient per year [64].

Bonnevie et al. conducted a systematic review and meta-analysis, which concluded that HFNC improved PaCO_2_, acute exacerbation, and QoL in patients with stable hypercapnic COPD in both short-term and long-term [66]. Although the improvement in PaCO_2_ reached statistical significance, it remains unclear whether it was a clinically important difference.

Nagata et al. conducted an RCT with 93 patients in 2022 [67]. They showed that HFNC + LTOT significantly reduced the number of moderate/severe exacerbations as compared to LTOT alone [67]. However, there was no statistically significant difference between HFNC + LTOT versus LTOT alone when looking at severe-only exacerbations [67]. HFNC also prolongs the time to the first moderate or severe exacerbation of COPD as compared to LTOT [67]. They further analyzed QoL using the SGRQ-C (compact version of SGRQ) looking at total score, symptoms score, activity score, and impact score. Only the total score at 24 weeks and the impact score at 12 weeks were significantly improved in the HFNC group, although the trend showed generally improved QoL across the board at 52 weeks [67]. They did not find any statistically significant differences between the two groups in terms of dyspnea as per the mMRC score, pH, PaO_2_, PaCO_2_, FVC, FEV1 DLCO, 6MWT distance, or all-cause mortality at 52 weeks. Resting SpO_2_ was significantly higher only at 52 weeks in the HFNC group [67].

Horvath et al. suggested that a subset of patients with chronic hypercapnic COPD who failed to benefit from NIV may have long-term reductions in PaCO_2_ with HFNC, although the clinical significance remains unclear due to data limitations [68].

McKinstry et al. focused on measuring transcutaneous carbon dioxide (ptCO_2_­) in stable hypercapnic COPD patients using NIV versus HFNC. They found that NIV was significantly better in reducing ptCO_2_ at 60 minutes than HFNC (mean: -5.3, SD: 5 mmHg versus mean: -2.5 SD: 3.5 mmHg, respectively, p = 0.021) [69]. However, there was no statistically significant difference between NIV and HFNC for ptCO_2_ reductions ≥ 4 mmHg and ≥8 mmHg [69]. HFNC was associated with significantly increased comfort, fit, and ease of application [69].

Huang et al. reviewed four RCTs, which looked at PaCO_2_ (primary outcome) and PaO_2_ (secondary outcome) in patients with hypercapnic COPD using HFNC versus conventional oxygen therapy, which included both domiciliary and acute settings [70]. There was no significant difference in either outcome between the groups [70]. However, the heterogeneity of the population (inclusion of both hypercapnic AECOPD and stable hypercapnic COPD patients) makes the interpretation difficult.

Overall, the limited literature suggests that home HFNC is beneficial in the management of stable hypercapnic COPD as it reduces moderate/severe exacerbations, is more comfortable, and improves QoL. A randomized controlled trial showed that further studies are needed to look more closely at the long-term effects of home HFNC on exercise tolerance, hospitalizations/admission-free survival, and mortality. A randomized control trial has demonstrated that home mechanical ventilation (domiciliary NIV) added to home oxygen therapy can improve admission-free survival and quality of life [71]. Future work could include a comparison of outcomes between home HFNC versus home NIV. Furthermore, with the advancement of telemonitoring technology, there may be scope to enhance compliance with domiciliary respiratory support therapies [72].

HFNC in the Palliative Care Setting

There is limited evidence on the efficacy of HFNC in symptom control in cancer patients. Epstein et al. evaluated the potential of HFNC as a management strategy for breathlessness in cancer patients’ breathlessness [73]. Most patients remained stable or improved, while a minority of the patients worsened [73,74]. The role of HFNC in managing breathlessness in those with established treatment limitations remains unclear [4,75]. Further work is needed to evaluate the effectiveness of HFNC in the management of acute respiratory failure with do-not intubate/do-not-resuscitate orders as compared to NIV, conventional oxygen therapy, and the use of opioid medication [4,75]. HFNC could be used as an adjunct to pharmacological therapy and other therapies in the management of patients with respiratory failure where mechanical ventilation has failed or is clinically inappropriate [4,76]. Isolated cases have shown great potential for HFNC in ameliorating symptom management in patients with life-limiting illnesses [77]. There is scant evidence to suggest that HFNC was comparable with NIV concerning improving dyspnea, O­_2_ saturation, and respiratory rate in patients with advanced cancer [78]. More work needs to be done comparing HFNC with conventional oxygen therapy, NIV, and medications in dying patients to further understand when to commence HFNC and how to adjust it, much like any other ventilation intervention in the palliative care setting [79].

Future Directions and Anticipation of Challenges

The vast majority of the studies of HFNC in the acute setting are in critical care. More work needs to be done to look at the feasibility and effectiveness of HFNC in medical wards, where nurse-to-patient ratios differ greatly from critical care. HFNC has the potential to be a simpler means of respiratory support due to the ease of application of the device and the need for fewer airway care interventions. With increasing familiarity with the technology and the incorporation of training on how to use HFNC, the ability of the multidisciplinary team to use HFNC effectively is likely to increase with time.

## Conclusions

HFNC has many clinical advantages over other therapies such as NIV, with enhanced patient comfort and ease of use. There are many potential applications of HFNC in the future for a wide variety of conditions resulting in respiratory failure. Further studies are needed, particularly in post-acute hospital admission and stable hypercapnic COPD patients, to assess the effects on acute exacerbation rate, QoL, and admission-free survival. This may be enhanced with the utilization of telemonitoring to improve compliance.
